# Mesenchymal Stem Cell Mechanisms of Action and Clinical Effects in Osteoarthritis: A Narrative Review

**DOI:** 10.3390/genes13060949

**Published:** 2022-05-26

**Authors:** Vilim Molnar, Eduard Pavelić, Kristijan Vrdoljak, Martin Čemerin, Emil Klarić, Vid Matišić, Roko Bjelica, Petar Brlek, Ivana Kovačić, Carlo Tremolada, Dragan Primorac

**Affiliations:** 1 St. Catherine Specialty Hospital, 10000 Zagreb, Croatia; vilim.molnar@svkatarina.hr (V.M.); eduard.pavelic@svkatarina.hr (E.P.); klaric1995@gmail.com (E.K.); vid.matisic@svkatarina.hr (V.M.); petar.brlek@svakatrina.hr (P.B.); 2 Faculty of Medicine, Josip Juraj Strossmayer University of Osijek, 31000 Osijek, Croatia; 3 School of Medicine, University of Zagreb, 10000 Zagreb, Croatia; kristijan.vrdoljak3@gmail.com (K.V.); martincemerin@gmail.com (M.Č.); 4 Department of Oral Surgery, School of Dental Medicine, University of Zagreb, 10000 Zagreb, Croatia; roko.bjelica@gmail.com; 5 General Hospital Karlovac, 47000 Karlovac, Croatia; ivana.kovacica@gmail.com; 6 Image Regenerative Clinic, 20121 Milan, Italy; carlo.tremolada@gmail.com; 7 Medical School, University of Split, 21000 Split, Croatia; 8 Faculty of Dental Medicine and Health, Josip Juraj Strossmayer University of Osijek, 31000 Osijek, Croatia; 9 Medical School, University of Rijeka, 51000 Rijeka, Croatia; 10 Medical School REGIOMED, 96450 Coburg, Germany; 11 Eberly College of Science, The Pennsylvania State University, University Park, PA 16802, USA; 12 The Henry C. Lee College of Criminal Justice and Forensic Sciences, University of New Haven, West Haven, CT 06516, USA

**Keywords:** mesenchymal stem cells, osteoarthritis, bone marrow, adipose tissue, placenta, stromal vascular fraction

## Abstract

With the insufficient satisfaction rates and high cost of operative treatment for osteoarthritis (OA), alternatives have been sought. Furthermore, the inability of current medications to arrest disease progression has led to rapidly growing clinical research relating to mesenchymal stem cells (MSCs). The availability and function of MSCs vary according to tissue source. The three primary sources include the placenta, bone marrow, and adipose tissue, all of which offer excellent safety profiles. The primary mechanisms of action are trophic and immunomodulatory effects, which prevent the further degradation of joints. However, the function and degree to which benefits are observed vary significantly based on the exosomes secreted by MSCs. Paracrine and autocrine mechanisms prevent cell apoptosis and tissue fibrosis, initiate angiogenesis, and stimulate mitosis via growth factors. MSCs have even been shown to exhibit antimicrobial effects. Clinical results incorporating clinical scores and objective radiological imaging have been promising, but a lack of standardization in isolating MSCs prevents their incorporation in current guidelines.

## 1. Introduction

Osteoarthritis (OA) is a common disease and the most common musculoskeletal progressive disorder for which appropriate non-surgical treatment has not yet been found [1]. The disease is characterized by the influence of proinflammatory cytokines, angiogenesis, chondrocyte hypertrophy, and apoptosis. This eventually leads to cartilage degeneration and changes in other tissues, such as subchondral bone, synovium, etc. Mesenchymal-stem-cell-based therapies (MSCs) have shown the greatest potential in halting disease progression, but the insufficient quality of research and evidence other than improving clinical parameters does not allow such therapies to enter the guidelines of international professional societies for the treatment of OA [2]. MSCs, depending on the environment in which they are located, secrete many factors, and their paracrine action leads to trophic, antimicrobial, immunomodulatory, and other effects [3,4,5]. The purpose of this review is to describe the proven effects of MSCs in OA through their studied effects in other tissues in order to identify the factors that could play a significant role in treating and slowing the progression of OA. Furthermore, a direct comparison through objectification, such as radiological changes and the use of scores regarding clinical results after the application of MSC-based therapies, was made.

## 2. Tissues Containing MSCs

There are three main tissues considered when harvesting stem cells with MSC potential: bone marrow, adipose tissue, and placenta. These donor sites are diverse enough to provide in vitro and in vivo results that differ significantly.

### 2.1. Bone Marrow

One of the most commonly used MSC extraction tissues is the bone marrow. It is primarily extracted from the posterior superior iliac spine, from where it has been proven to yield the most connective-tissue progenitor cells [6]. The Food and Drug Administration (FDA) has strict regulations on human cells, tissues, and cellular and tissue-based products (HCT/P) [7]. Therefore, in the United States, this technique is preferred and approved by the FDA as a minimally manipulated product for homologous use only [8]. In 1987, Friedenstein et al. proved that certain bone MSCs have different potentials for proliferation [9]. This was later elaborated on by a study that showed that CD56+ MSCs collected from the femoral shaft during total hip arthroplasty had a higher replicative rate than CD56- MSCs. They also proved that these cells tended to have a higher propensity for chondrocyte differentiation [10]. Other studies have compared the chondrogenic potentials of BM-MSCs and AD-MSCs and concluded that both MSC isolates can differentiate into chondrocytes in vitro [11,12,13,14]. A discernible difference becomes apparent when shifting to a higher cellular density well-plating, where BM-MSCs responded with improved chondrogenesis [15]. Similarly, other studies have also concluded that BM-MSCs have a stronger propensity to differentiate into chondrocytes in vitro [16]. An interesting study by Sivasubramaniyan et al. showed the difference in chondrocyte potential of BM-MSCs extracted from the femur by rasping to be greater than from traditional aspiration [17]. Hence, there exist location-specific differences that should be included when considering BM-MSCs as a therapeutic option.

### 2.2. Adipose Tissue

Another valuable source of MSCs is adipose tissue, known as adipose-derived MSCs (AD-MSCs), most often sourced from the abdominal, gluteal, or lumbosacral regions. The most common way of extracting adipose tissue is by lipoaspiration from subcutaneous abdominal tissue. From this lipoaspirate, MSCs are isolated by mechanical and then enzymatic degradation. These cells are then purified, filtered, and finally, cultured. Phenotypic analysis of AD-MSCs and BM-MSCs found they express: CD29, CD44, CD71, CD90, CD105/SH2, and SH3, which, together with SH2, are all considered to be markers for MSCs [11,18]. BM-MSC large quantity harvesting has shown that larger volumes of bone marrow aspirates may be contaminated with peripheral blood. AD-MSCs offer a better alternative with significantly less contamination. Due to their cellular buoyancy, they are easily extracted and provide a cellular yield 500 times higher than the equivalent amount of BM-MSCs per gram of tissue [14,19,20,21,22]. A further advantage of AD-MSCs is the associated harvesting technique. For example, harvesting large volumes of BM-MSCs is associated with increased donor-site morbidity [23,24]. Different buffers and culturing techniques can result in yield differences in AD-MSCs [25,26]. In general, AD-MSCs are metabolically active for longer durations, making them able to go through more replication cycles than BM-MSCs; however, no proposed mechanism has been given for this observation [13,27,28,29]. Further studies have shown that AD-MSCs offer an anti-inflammatory effect, which, in turn, can help regulate pain after injection [30,31]. The immunomodulatory effects of AD-MSCs seem to be stronger than those of BM-MSCs, with a stronger reduction in B-cell differentiation into immunoglobulin-producing cells [32,33,34]. BM-MSCs have also been shown to have a weaker anti-inflammatory effect, as evidenced by increased levels of tumor necrosis factor α (TNF-α) and transforming growth factor-β (TGF-β) [35]. Furthermore, AD-MSCs appear to have a paracrine influence, which, in turn, increases the formation of endothelial structures, altering the vasculature of the tissue [36,37]. The potential to differentiate into chondrogenic cells in combination with the immunomodulatory and paracrine effects make AD-MSCs a diverse product for clinical use that can result in marked clinical improvement.

### 2.3. Placenta

Besides bone marrow and adipose tissue, the placenta is the third most-used tissue for MSC gathering. MSCs are cells that, as first shown by Fukuchi et al., have multilineage potential. They are sourced from different layers of the placenta. These include the amniotic membrane (AM), along with a separate division of cells, such as the epithelial cells (AEC), amniotic fluid (AF), chorionic plate (CP), and umbilical cord (UC). The exact location from where MSCs can be isolated in placental extraction can result in different doubling times in vitro; as such, a rigorous and precise extraction method should be employed. Different extraction locations of placental stem cells can also result in different characteristics of cells [38,39]. These distinctive characteristics make isolating the exact layer for placental extraction a vital component in the therapeutic application. As first theorized and later proven by Hayflick and Moorhead, after a set number of divisions, human cells lose their mitotic potential; however, embryonic cells exhibit a higher limit of divisions before entering senescence [40,41]. Cellular senescence at the cartilaginous level has been proven by evaluating B-galactosidase and H-thymidine levels, which measure senescence and mitotic activity, respectively, and are proportional to the age of donors [42]. Yoon et al., after 3 weeks of culturing AF-MSCs with a chondrocyte-inducing medium, conducted an RT-PCR analysis of chondrogenic gene expression, which showed upregulation of aggrecan and type II collagen, demonstrating that AF-MSCs are capable of differentiation into chondrogenic lineages. They also examined the soluble factors and found increased production of TNF-α, VEGF, TGF-β, Leptin, IL-8, and IL-6 [43]. Chorionic and umbilical-cord-derived MSCs (C-MSC and UC-MSC, respectively) have been proven to have a higher expression of CD56 markers when compared to BM-MSC, and therefore increased clonogenic and differentiation potentials. They also concluded, through flow cytometry analysis of UC-MSC, C-MSC, and decidual-derived MSC, that UC-MSC and C-MSC have a lower proliferation percentage for CD45 and CD3, but exhibit higher suppression of T cells in vitro [44]. Further, UC-MSCs were shown to have the fastest population-doubling time. While cells of embryonic origin are an excellent source of MSCs, they show drastically different properties regarding immunogenicity, proliferation, and trophic effects with respect to the source. Therefore, one must carefully isolate the cell layers to obtain consistent results.

## 3. Mechanism of Action

### 3.1. MSC

**Figure 1:**
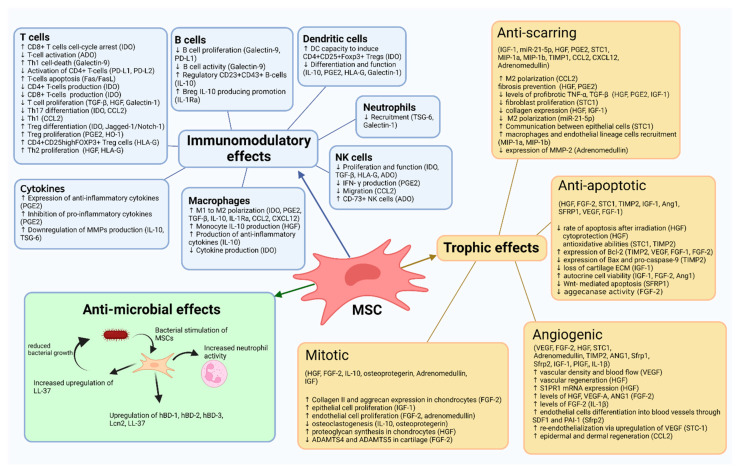
A summary of mesenchymal stem cell (MSC) effects. IDO—Indoleamine 2,3-dioxygenase; ADO—adenosine; PD-L1—programmed cell death ligand 1; PD-L2—programmed cell death ligand 2; TGF-β—transforming growth factor β; HGF—hepatocyte growth factor; CCL2—monocyte chemoattractant protein 1 (MCP-1); PGE2—prostaglandin E2; HO-1—heme oxygenase-1; HLA-G—human leukocyte antigen-G; IL-10—interleukin 10; TSG-6—tumor necrosis factor-stimulated gene 6; IL-1Ra—interleukin 1 receptor antagonist; DC—dendritic cells; IFN-γ—interferon-γ; HBD—human b-defensins; lcn2—lipocain family; FGF—fibroblast growth factor; IGF—insulin growth factor; VEGF—vascular endothelial growth factor; STC1—stanniocalcin-1; TIMP2—tissue inhibitor of metalloproteinases 2; ADAMTS—a disintegrin and metalloproteinase with thrombospondin motifs; SDF—stromal cell-derived factor; SFRP2—secreted fizzled related protein. Created with BioRender.com.

Depending on the environment in which they are located, MSCs secrete numerous factors and thus act paracrine and autocrine, performing trophic, immunomodulatory, and antimicrobial effects [3,4,5]. A summary of the effects is shown in Figure 1.

#### 3.1.1. Trophic Effects

Trophic effects originate from cells as bioactive factors that primarily affect neighboring cells. These effects are most commonly classified into anti-apoptotic, anti-scarring, angiogenic, and mitotic effects in order to regenerate tissue properties with the resulting gain of coordinated function [45].

Paracrine-acting molecules often play a role in two or more of these trophic effect categories [46]. 

It is important to keep in mind that the trophic effects of MSCs may differ due to several factors, such as donor specificity (autologous, allogeneic, xenogeneic), tissue origin, separation from other cell types/preparation methods, exhibited cell characteristics associated with behavior, and delivery site [47].

##### 3.1.1.1. Anti-Apoptotic Effects

Anti-apoptotic effects of MSCs were demonstrated in the work by Wang et al., in which the apoptosis rate was reduced following the application of BM-MSCs and separately genetically modified MSCs overexpressing hepatocyte growth factor (HGF, MSC-HGF) in an induced lung-irradiation model [48]. This suggests both an anti-apoptotic effect of HGF secreted from MSCs as well as by the MSCs alone. Furthermore, the reduced cytoprotective ability in HGF-knockdown human BM-MSCs indicates that sustained production of HGF may play an important role in anti-apoptotic cytoprotection in elastase-exposed lungs [49]. Further suggesting that HGF plays an important anti-apoptotic role in cardiac injury, BM-MSCs primed through genetically engineered HGF exhibited improved cell viability, which resulted in enhanced vascular regeneration and restored cardiac function to hearts that had suffered from myocardial infarction [50]. 

In vitro examination revealed that paracrine effects of BM-MSC-secreted stanniocalcin-1 (STC1) mediate anti-apoptotic effects in both UV-irradiated fibroblasts and hypoxia-induced injury of lung cancer epithelial cells [51]. STC1 is more highly expressed in MSCs from human palatine tonsil (T-MSC) compared to MSCs from bone marrow or adipose tissue, in which it mediates critical antioxidative abilities [52]. 

It was found that by activating the pro-survival Akt/SFRP2 pathway, the exosomes derived from tissue matrix metalloproteinase inhibitor 2 (TIMP2)-overexpressing UC-MSC increased the in situ expression of anti-apoptotic Bcl-2 and decreased that of proapoptotic Bax and pro-caspase-9 in infarcted myocardium. Furthermore, UC-MSCs showed an anti-oxidative effect by upregulating superoxide dismutase (SOD) as well as glutathione (GSH) and decreasing the malondialdehyde (MDA) level in MI models [53].

Potentially important for orthopedic applications, the autocrine MSC secretion of TGF-β may prevent their apoptosis. Following the pathway of growth plate chondrocytes, MSCs become hypertrophic despite chondrogenic differentiation and eventually undergo apoptosis. Contrary to pro-hypertrophic BMP signaling, TGF-β signaling inhibits hypertrophy in vitro and in vivo [54]. Whether and how autocrine MSC-TGF-β effects can reduce apoptosis through reduction of hypertrophy remains to be examined. It has been demonstrated that specific physical conditioning regulates the hypertrophic effects in achieving in vitro MSC chondrogenesis. Namely, compared to free-swelling controls, a deferral dynamic compression enhanced cartilage formation and suppressed chondrocyte hypertrophy, accompanied by the activation of differentiation and proliferative pathways and suppression of regulatory pathways [55].

An important angiogenic factor secreted by MSCs, VEGF, promotes cell survival by inducing the expression of anti-apoptotic molecules such as Bcl-2. Hence, reports have suggested that Bcl-2 mediates the regulation of chondrocyte apoptosis in OA, as well as the regulation of matrix gene expression through Sox9. Evidence also shows higher levels of VEGF expression in OA chondrocytes than in non-arthritic chondrocytes [56]. However, these data require more research and remain controversial. Interestingly, to emphasize the importance of secreted VEGF from MSCs, synovial VEGF may be involved in pain pathways in knee OA [57]. Synovial membrane cell culturing has shown that VEGF has a stimulating effect on apelin mRNA and protein expression. Apelin is known to be a major OA-related adipokine, and it was found to enhance IL-1β expression in human OA synovial fibroblasts [58]. Increased VEGF values have been associated with higher VAS values; therefore, increased apelin could contribute to an increased perceived level of pain.

Another factor that acts on anti-apoptotic mechanisms is insulin growth factor-1 (IGF-1) through a tyrosine-kinase survival pathway [59]. IGF-1 reduces the loss of chondrocytes and matrix integrity but also has an anabolic role in OA [60]. In a murine OA model, analysis of knee OA joints 4 and 8 weeks after OA induction suggested IGF-1-mRNA genetically engineered AD-MSCs had a better therapeutic effect over native AD-MSCs by having a lower histological OARSI score and less loss of cartilage extracellular matrix. IGF-1-AD-MSCs increased type II collagen and aggrecan expression in chondrocytes in the inflammatory environment compared to untreated AD-MSCs [61]. A further study indicated the therapeutic potential of MSC-IGF-1 pro-regenerative actions to cardiac and skeletal muscles, namely in a mouse model of chronic Chagas disease [62]. Taken together, this implies that IGF-1 is an important paracrine trophic effector molecule of MSCs in OA treatment. Another important effect of IGF-1 is thought to be an autocrine function. This was studied in human UC-MSC, in which IGF-1 may be capable of influencing cell viability, presumably by activation of the Akt/GSK-3β signaling pathway [63].

FGF2 is also an important molecule secreted by MSCs that is involved in multiple paracrine activities and can exert anti-apoptotic effects. The autocrine activity of MSC-secreted FGF has been reported under stress conditions, in which FGF1 and FGF2 translocate via the endosomal membrane into the cytosol and nucleus, where they mediate their anti-apoptotic activity by increasing the expression of Bcl2 [64]. FGF2 demonstrates higher anti-apoptotic activity than FGF1, which may be due to FGF2 remaining in the nucleus longer than FGF1 due to a lack of phosphorylation [65]. Regarding OA, FGF-2 has been found to be an endogenous chondroprotective agent in articular cartilage by inhibiting aggrecanase activity [66].

Studies also report that MSCs can synthesize and secrete granulocyte-macrophage colony-stimulating factor (GM-CSF) under hypoxic conditions. Since this secretion occurs together with the anti-apoptotic factor VEGF, it is suggested that GM-CSF may also be relevant in the inhibition of cellular apoptosis and restoring tissue homeostasis [67]. 

A study that examined the effects of induced expression of secreted frizzled-related protein 1 (SFRP1) in the myocardium found that it attenuated cardiac dysfunction by inhibiting Wnt-signaling-pathway-activation-mediated apoptosis. Since SFRP1 is expressed in mesenchymal cells, MSCs secreting SFRP1 might mediate anti-apoptotic mechanisms [68]. The interaction of SFRP1 and its Wnt antagonistic activity may also play a role in treating OA pathology [69].

The observed anti-apoptotic molecules, and other molecules yet to be discovered, secreted by MSCs have potential therapeutic effects in numerous tissues along with in OA pathology. However, it is to be recognized that, while a great amount of work is being done with MSCs, orthopedic applications are limited. That is why translational studies and inferencing must be applied for further orthopedic applications.

##### 3.1.1.2. Anti-Scarring Effects

Repeated injuries, chronic inflammation, and repair are all scenarios that are susceptible to fibrosis. This dysfunctional tissue architecture is characterized by excessive accumulation of extracellular matrix components, such as the collagen produced by fibroblasts [70]. The process is initiated when immune cells such as macrophages release soluble factors that stimulate fibroblasts. MSCs act through paracrine signaling pathways that differentiate these cells and induce others to alter their functions to reduce the scarring effect. 

To understand anti-scarring (anti-fibrosing) effects in OA, first, it should be mentioned where fibrosis occurs during the progression of the disease. Two main locations seem to play a role in the pathogenesis of OA: the synovium and cartilage [71]. When the articular cartilage is injured, the chondrocytes’ response to stress causes them to proliferate abnormally, leading to their de-differentiation into fibroblast-like fibrotic chondrocytes. This subset of chondrocytes secretes different extracellular matrix (ECM) proteins than normal hyaline-cartilaginous chondrocytes. Mainly, they secrete collagen type I instead of collagen type II or aggrecan [72]. Cartilage formed by these chondrocytes is stiffer and possesses different biomechanical properties, becoming more similar to fibrocartilage, which is pathognomonic for degenerative OA [73]. 

The second important tissue that is affected by fibrosis during the pathophysiologic course of OA is the synovial membrane. This is due to synovitis, which results in infiltration of inflammatory cells, hyperplasia of the synovial membrane cells, increased stromal vascularization, and fibrosis of the sub-lining synovial cells [74]. Synovium that is affected by fibrosis gives rise to excessive proliferation of fibroblasts and disturbs the balance between collagen synthesis and catabolism [75]. 

Since there is limited information about the anti-scarring effects of MSCs in OA pathogenesis, it is necessary to observe mechanisms of MSC action in other pathologies as well. AD-MSC exosomes have been shown to ameliorate cardiac damage after MI by activating S1P/SK1/S1PR1 signaling and promoting macrophage M2 polarization [76]. M2 macrophages resolve inflammation and help tissue healing through tissue development and turnover, metabolism, and endocrine signaling [77]. A study by Li et al. examined the apoptotic bodies of AD-MSCs and found abundant expression of microRNA (miRNA)-21-5p, which was found to target Kruppel-like factor 6 (KLF6). The macrophages overexpressing KLF6 were inhibited to undergo polarization to the M2 phenotype; therefore, targeting KLF6 with miR-21-5p resulted in augmented skin-wound healing [78]. 

A study demonstrated that infusion of AD-MSCs prevented fibrosis formation in irradiated rat lungs, preserving their functional architecture [79]. Furthermore, secretions of anti-fibrotic factors such as HGF and prostaglandin E2 (PGE2), which have been associated with ameliorating lung fibrosis, were elevated compared with those of the control group [80,81]. Moreover, in a separate study conducted by Cahill et al., it was detected that HGF knockdown MSCs were unable to protect against pulmonary fibrosis in a murine model [82]. In contrast, pro-fibrotic mediator levels of TNF-α and TGF-β1 were decreased after infusion with AD-MSCs [79].

One of the molecules that has shown anti-scarring effects and that is secreted by MSCs is STC1. As shown by Ono et al., through STC1 secretion, MSCs obtain anti-fibrosing effects that can correct the inadequate communication between epithelial and mesenchymal cells, consequently ameliorating idiopathic pulmonary fibrosis [83]. STC1 secretion is increased only under specific cell-stress conditions, namely, changes in actin stress fibers and actin-myosin tension [84]. Since OA involves inappropriate mechanical loading mitigating cellular stress, MSC-secreted STC-1 could play an important therapeutic role. 

Macrophages have a crucial role in wound healing. They release a cytokine called macrophage inflammatory protein-1α (MIP-1α), which is the ligand of a chemokine CC receptor 1 (CCR) expressed on the MSCs’ surface. In this way, macrophages recruit MSCs to the site of inflammation [85]. Chen et al. showed that BM-MSCs express a great amount of chemoattractant MIP-1α and MIP-1b, suggesting that factors released by BM-MSCs recruit macrophages and endothelial lineage cells into the wound, thus enhancing wound healing [86]. 

MMP-2 levels are increased in OA, and another study found that its expression is reduced by adrenomedullin [87]. Furthermore, adrenomedullin was shown to have increased serum levels in knee OA patients [88]. Its secretion from MSCs has been examined in studies that used recombinant gene technology to enhance its expression in MSCs. One of these studies showed significantly improved heart function and a decreased percentage of fibrotic area and expression of MMP-2. Moreover, the expression of collagen I and III together with fibrosis-related genes (such as *ADM*) also decreased in the ADM-MSC-treated group [89]. 

It was demonstrated that overexpression of another factor, SFRP2, from MSCs enhanced the effect of bone marrow macrophage-derived conditioned media upon wound healing [90]. However, the analysis showed significant upregulation of SFRP2 as a pro-fibrotic marker occurring in the infrapatellar fat pad of a murine model of OA associated with synovial fibrosis [91,92]. SFRP2 seems to be an important part of the anti-scarring MSC secretome; however, its therapeutic properties remain unclear in the case of reducing fibrosis in the OA joint after MSC application.

BM-MSC-derived exosomes were shown to improve DM-induced myocardial injury and fibrosis via inhibition of the TGF-Î^2^1/Smad2 signaling pathway [93]. Further, BM-MSCs may inhibit the TGFβ-1/SMADs pathway, studied in a mouse model of CCL4-induced liver fibrosis [94]. Since mechanical-stress-induced overexpression of TGF-β1 from osteoclasts is responsible for chondrocyte apoptosis and cartilage degeneration in OA, MSCs anti-TGF-β1 actions may play an important role in MSC treatment of OA [95].

Another molecule that showed anti-fibrotic effects is IGF-1. Skeletal muscle sections of both MSC and MSC-IGF-1-treated mice showed a reduction in fibrosis compared to saline controls mediated by IGF-1 secreted from MSCs [62]. Similarly, mice with burn injuries displayed increased wound contraction, healing rates, reduced collagen disposition, and inflammatory infiltration following treatment with IGF-1-expressing placenta-derived MSCs. IGF-1 from MSCs also increased VEGF expression and decreased TGF-β1, collagen I, and collagen III expressions in vivo, suggesting an anti-fibrotic effect [96]. 

Many of the aforementioned effector molecules mediate anti-fibrotic actions. However, the questions remain regarding which tissue origin of MSCs produces the best therapeutic effect; and how, regarding which pathology, when, and by which interactions with other paracrine effects it occurs. Of the latter, angiogenic modulation seems to be important, since the tissue needs to maintain its viability if it is to avoid fibrotic replacement.

##### 3.1.1.3. Angiogenic Effects

Angiogenesis is a process in which new blood vessels emerge from preexisting ones [97,98]. The trophic, vasculature-forming effects of MSCs have been documented. They have also shown differences in paracrine effects, as evidenced by the differing secretion of PGE2 and TGF-β1, found to have higher expression in AM-MSCs, while HGF and VCAM-1 are present in C-MSCs. Finally, D-MSCs showed the highest secretion of Ang-1 and VEGF and the lowest secretion of TGF-β1, while UC-MSCs showed the highest secretion of IGF-1 [39]. It has been shown that BM-MSCs implanted into ischemic myocardium promoted production of VEGF, increased vascular density and blood flow, and decreased apoptosis, all of which were likely influenced by the secretion of bioactive molecules, while some MSCs also differentiated directly into endothelial cells [99].

S1PR1 has an important role in regulating endothelial cell cytoskeletal structure, migration, capillary-like network formation, and vascular maturation [100]. A study suggested that MSC-secreted HGF may play an angiogenic trophic role via S1PR1. Compared with the unmodified-BM-MSC and radiation groups, BM-MSCs-HGF increased S1PR1 mRNA expression on days 28 and 180 after radiation [48]. Genetically engineered BM-MSC-HGF in MI hearts improved vasculogenic ability, with enhanced vascular regeneration and restored cardiac function [50]. More research should be carried out to determine when/whether MSCs may use the S1PR1 signaling pathway.

The expression of FGF-2 mRNA by MSCs and its intracellular protein presence have been documented to benefit MSCs’ angiogenic effect. FGF-2 appeared to be more potent than VEGF in inducing human umbilical vein endothelial cell proliferation [101]. Investigations of FGF-2 production showed increased secretion of VEGF-A, HGF, bFGF, and ANG-1 from amniotic-derived MSCs compared to AD-MSCs [102]. Nevertheless, supporting the long-term angiogenic efficacy of AD-MSCs in ischemic mouse tissues, FGF-2 modulates angiogenesis via an autocrine mechanism, and together with VEGF has a potent synergistic effect on the induction of angiogenesis in vivo [103]. Concerning chondrocyte angiogenesis, IL-1β stimulation of murine chondrocyte cells (ATDC5) increased FGF-2 expression and promoted endothelial progenitor cell tube formation and migration. Corroborating this finding, FGF-2-neutralizing antibodies abolished ATDC5-conditional medium-mediated angiogenesis in vitro, as well as its angiogenic effects in in vivo models [104]. 

Additional angiogenic benefits of MSC-contained factors have been shown in the work by Ni et al., Human UC-MSCs overexpressing TIMP2 significantly improved in vivo cardiac function and promoted angiogenesis in MI injury, possibly via the Akt/SFRP2 pathway [53]. A study by Sun et al. found that BM-MSCs secrete SFRP2, which in turn induces macrophages to secrete SDF1 and plasminogen activator inhibitor-1 (PAI-1). Both SDF1 and PAI-1 promote endothelial cells to differentiate into blood vessels [90]. The work of Sillat et al., explored the differentiation of MSCs into adipogenic lineage and basement membrane formation of the perivascular milieu; they found increased expression of MMP-9, TIMP-2, and collagen type IV, a crucial component in endothelial cell basement membranes. As such, the balance between MMP-9 and TIMP-2 could play a crucial role in angiogenesis [105].

Studies conducted on the trophic effector STC1 have also been shown to mediate angiogenic effects through the process of STC-1-regulated VEGF expression, which is mediated via PKCβII and ERK1/2 [106]. Other research found that re-endothelialization by exosomes from AD-MSCs on post-injury carotid endarterium could be enhanced by genetic modification of the exosomes to contain elevated STC-1 levels [107]. Work done by Bhandi et al. showed that hypoxic conditions affect the secretory profile of dental pulp stem cells. They found an increase in angiogenic cytokines, which is expected with upregulation of HIF 1α. Growth factors such as EPO, Ang -2, bFGF, SCF, TGFα, and VEGF were increased [108].

Another study indicates that autophagy in MSCs drives the paracrine secretion of VEGF by directly phosphorylating ERK. VEGF further acts in cutaneous wound healing by alleviating vascularization deficiency by promoting angiogenesis [109]. More specifically, it mediates the differentiation of endothelial progenitor cells into endothelial cells via paracrine mechanisms [110].

It is suggested that another factor secreted from MSCs might potentially be effective in promoting therapeutic angiogenesis/arteriogenesis. In vitro, SFRP1 increases platelet-derived growth factor-BB expression in MSC and enhances β-catenin-dependent cell-cell contacts between MSCs themselves and endothelial cells or smooth muscle cells. In vivo, SFRP1 increases MSCs’ functional integration around newly formed vessels and vessel maturation through a glycogen synthase kinase 3 β-dependent pathway [111].

It has been shown that BM-MSCs genetically modified to secrete more angiopoietin-1 (ANG-1) had both a protective and an alleviating therapeutic effect on pulmonary vascular endothelial permeability caused by induced acute lung injury [112,113]. ANG-1-overexpressing BM-MSCs were found to promote wound healing with increased epidermal and dermal regeneration and enhanced angiogenesis. Non-modified MSCs had less effect but still helped compared to control [114]. When compared, MSCs derived from bone marrow, adipose tissue, and Wharton’s jelly (umbilical cord) all showed similar ANG-1 concentrations in their supernatants [115].

MSC-induced accelerated wound healing requires MSC secretion of CCL2 (also known as monocyte chemoattractant protein 1–MCP-1). BM-MSC isolated from CCL2 deficient mice was unable to repolarize macrophages towards a reparative phenotype to the same extent as wild-type, and this was accompanied by reduced angiogenesis and re-epithelialization [116]. Further, it was shown that BM-MSC-secreted CCL2 and CXCL12 synergic effects induce M2 polarization [117]. Moreover, it was demonstrated that AD-MSCs secretome contains CCL2 [118]. 

An interesting paracrine angiogenic interaction between BM-MSC-secreted IL-6, colorectal cancer-secreted endothelin-1 (ET-1), and endothelial cells has been reported. The secretion of IL-6 from MSCs enhanced the secretion of ET-1 in cancer cells, which promoted the activation of Akt and ERK in endothelial cells, thereby enhancing their capabilities for tumor angiogenesis [119]. In a skin-flap ischemia/reperfusion injury model, extracellular vesicles from AD-MSCs were found to increase flap recovery and capillary density (increased tube formation) via increased IL-6 expression through the classic signaling pathway [120]. 

Despite the fact that angiogenesis is one of the main processes that drive inflammation in the pathophysiologic course of knee OA, it has yet to be determined how it can be used to help tissue healing. Such a seemingly paradoxical effect can theoretically be explained by the change in the microenvironment mediated by MSCs. Regardless of the different MSCs’ origin, high levels of IL-6 are usually constantly retrieved (AD-MSCs/BM-MSCs) [121]. Further emphasizing angiogenic paracrine activity through secreted molecules, when neutralizing antibodies against IL-6 (also VEGF and MCP-1) are used, angiogenesis is significantly impaired [122]. Since multiple sources report that IL-6 is partly responsible for OA progression by inducing angiogenesis, and therapeutics that block IL-6 mediated signaling inhibit angiogenesis in arthritis, MSCs’ therapeutic potential in treating OA needs to be clarified: whether and how secreted IL-6 interacts with vessel formation in OA when MSCs are implanted [123,124]. However, the notion exists that IL-6 may be an important regulatory molecule in both physiologic and pathological angiogenesis [125]. Since MSCs relieve OA, we hypothesize that MSC-regulated IL-6 secretion may cause optimization of IL-6 signaling when applied to the OA joint.

A study has suggested that blocking critical anti-angiogenic secretome molecules could be used to enhance cartilage regeneration and repair. Indian Hedgehog (IHH) and Serpin E1 are uniquely upregulated factors during chondrogenesis and endochondral ossification; they act in an anti-angiogenic fashion in this context. Chondrogenically differentiated BM-MSCs showed increased endothelial cell proliferation when IHH and Serpin E1 were blocked in a conditioned medium. Moreover, endothelial migration was further increased by IHH and Serpin E1 blocking. This knowledge could be useful in articular cartilage repair [126].

MSC transplantation on scaffolds has been used to treat traumatic chondral and osteochondral lesions with good results both in vitro and in vivo [127]. The process of filling in the cartilage defects with new tissue is where MSCs excel, one of the reasons probably being angiogenesis, which promotes tissue integration. The addition of allogenic MSCs to an autologous chondron matrix showed no allogenic DNA in the regenerated tissue, indicating allogenic MSCs were acting as paracrine modulators to promote tissue healing [128].

The angiogenic modulation mediated by the MSC secretome offers insights that differ regarding the underlying mechanisms, which remain under-investigated. Hence, a vast therapeutic potential may be hidden behind gaining a better understanding of angiogenesis and the pathways involved in OA. OA pathogenesis lacks angiogenesis regulation [129]. Thus, MSCs could put in place positive and negative feedback mechanisms that could slow disease progression. However, further research into the modulation of such effects must proceed with caution, as the introduction of MSCs to regulate angiogenesis may further degenerate the joint and could potentially expedite OA progression. When apoptosis is prevented, fibrosis contained, and angiogenesis optimally modulated, mitotic action remains to exert further trophic action of the MSC therapy.

##### 3.1.1.4. Mitotic Effects

Mitosis is a biological process in the cell cycle and, when not physiologically regulated, may generate pathologic conditions. There are several ways this may occur, mainly mutations/variations of the genes that encode regulatory proteins and exogenous/endogenous stimulation or stress. Moreover, it is important to distinguish the existence of pro-mitotic growth factors and anti-mitotic tumor suppressor proteins and the effects of various stimuli that may lead to these conditions [130,131]. Adult stem cells provide a system to replace naturally expiring tissue cells in order to provide physiological balance in the organism. This makes the organism capable of slowly changing its properties as a function of age and/or use [45].

BM-MSCs were found to express osteoclastogenesis-suppressing cytokines: IL-10 and osteoprotegerin [132]. Evidence demonstrated that macrophage-derived IL-10 regulated the expression of the osteogenic markers RUNX2, COL1A1, and ALPL. Factors released by anti-inflammatory macrophages enhanced MSC osteogenic activity as well as cell migration, while the factors secreted by pro-inflammatory macrophages substantially increased MSC attachment and migration [133].

Exosomes represent a recently characterized cell-to-cell communication system that transports important factors such as nonsecretory proteins, RNAs, lipids, and metabolites. Proteomic data analysis found that exosomes derived from primed BM-MSC are packaged with elevated levels of extracellular and plasma membrane-associated proteins. Fibronectin was the most abundant protein detected, and data established that it mediated the mitogenic properties using a specific inhibitor of AKT signaling, blocking phosphorylation of proteins downstream in the fibronectin signaling cascade [134]. 

A suggestion has been made that HGF, one of the MSC-secreting factors, has the potential to promote chondrocyte survival and the proliferation of articular cartilage. In response to HGF, increases in cell proliferation and total cell number in vitro were reported in isolated immature rabbit and rat chondrocytes. In addition, HGF stimulated proteoglycan synthesis in chondrocytes. An investigation of articular cartilage repair using HGF in vivo was made by observing the animals for 6 months after HGF was injected into osteochondral-damaged rabbit knee joints. HGF effectively repaired osteochondral defects [135]. 

It has been reported that BM-MSCs contain a major depot of active FGF-2, which is released upon cell injury. It was shown that released FGF-2 is capable of inducing mitogenic action in animal models by acutely stimulating neuropoiesis and inducing concentration-dependent proliferation of rat cortical neural progenitor cells [101]. However, this factor remains disputed, as several findings demonstrated that FGF-2 shows contradictory effects in OA, namely in different species [136]. Firstly, FGF-2 might induce catabolic effects on human OA cartilage. It is released from damaged cartilage into the synovial fluid of OA patients and activates the extracellular-signal-regulated kinase signaling pathway. FGF-2 has a negative impact on proteoglycan content in cartilage explants and articular chondrocytes through RUNX2 and ADAMTS5 activation. FGF-2 is positively correlated with MMP1 and MMP13, but negatively with aggrecan and collagen II in human OA chondrocytes [136]. Another study compared FGF-2’s effects in OA as well. Catabolic effects of FGF-2 were found in the regulation of the transcription of MMP1, MMP13, and ADAMTS5, ultimately promoting cartilage degradation. It also functions as a chondrocyte mechanotransducer [137].

On the other hand, the role of FGF-2 in murine cartilage shows different findings, suggesting that FGF-2 plays a chondroprotective role and may have potential as a cartilage repair agent. Ablation of FGF-2 leads to accelerated aging and surgically induced OA development in mice. Furthermore, FGF receptor antagonists downregulate the expression of MMP1 and MMP13 in human OA chondrocytes [136]. Moreover, FGF-2 is an effective agent for enhancing the proliferation and chondrogenesis of human synovium-derived stem cells [138]. This correlates with the positive effects mediated by FGF-2 being released from MSC after synovial implantation and injury. The main anti-catabolic effects in cartilage are (1) the induction of TIMP1 expression, which encodes an inhibitor of MMPs; and (2) suppression of IL-1-induced expression and activity of ADAMTS4 and ADAMTS5 [137]. This may be due to the activation of both FGFR1 and FGFR3, thus exerting catabolic and anabolic effects, respectively, on cartilage maintenance. As such, FGFs have a biphasic effect that is concentration-dependent. This biphasic effect via FGF can be potentially linked by research conducted on the ratio of MMP/TIMP. An increased MMP/TIMP ratio has been found in the microenvironment around BM-MSCs in the work by Grit et al. The authors concluded that this environment could help direct MSC differentiation and the degree of proliferation [139]. Furthermore, the work done by Lian et al. found an increase in type II collagen while MSCs underwent chondrogenic differentiation. Type II collagen has been found to suppress chondrocyte hypertrophy [140]. Inferring from the latter work by Gritt and colleagues, it is plausible to hypothesize that the degradation of cartilage by MMPs could act as a catalyst to stimulate the MSCs into non-hypertrophic chondrocyte phenotypes, thus restoring cartilage homeostasis.

Although studies have shown a potent anti-scarring effect by the previously mentioned peptide, adrenomedullin, it also plays a role in growth and mitogenesis, promoting endothelial cell growth and survival through activation of MAPK/ERK downstream signaling pathways [141].

MSCs have also shown their ability to express a Wnt antagonist, SFRP1 [142]. In mouse strains with OA, SFRP1 expression was reduced in adult articular chondrocytes. This can be linked to the development of OA through the recent findings of elevated Wnt signaling that renders adult articular chondrocytes prone to premature aging and cell death [143]. It was found that the Wnt pathway is one of the critical signaling pathways that are key regulators and activators of cellular and molecular processes during OA development. Wnt signaling molecules and regulators were shown to be abnormally activated or suppressed. This implies that SFRP1 secreted from MSCs, as a Wnt antagonist, might be an important factor in treating OA [69].

MSCs can also act via extracellular vesicles to upregulate SMAD6 expression by transporting TGF-β. SMAD6 is an inhibitory Smad that regulates signaling downstream of type I TGF-β [144]. Other research found that TGF-β stimulated chondrogenesis in OA chondrocytes. Inhibiting TGF-βR1 suppressed MMP-13 in OA-MSC but stimulated it in OA chondrocytes. This diversity is hypothesized to arise from the difference in high and very low ALK5 expression in OA chondrocytes and OA-MSC, respectively [145]. Hence, the characteristics of implanted MSCs need to be studied more with regard to TGF-β signaling to avoid possible catabolic effects in local OA.

TGF-β1 is activated in the subchondral bone in response to altered mechanical loading in OA. High concentrations of TGF-β1, which is activated in subchondral bone during OA pathogenesis, lead to the formation of marrow osteoid islets accompanied by high levels of angiogenesis [146]. Since it is associated with both OA and MSCs’ therapeutic effects, it requires detailed examination. Data suggest that TGF-β could exert a biphasic, dose-dependent effect on MSC proliferation. TGF-β on one side promotes the proliferation and differentiation of MSCs into osteoprogenitors, cardiac and smooth muscle cells, tenocytes, and chondrocytes. However, TGF-β can also inhibit MSC differentiation into myoblasts, osteocytes, and adipocytes [147]. Therefore, further detailed analysis must be derived to arrive at a generalized conclusion for TGF-β as a mitogenic factor. 

Few studies have reported the effects of IGF-1 on promoting the growth, survival, self-renewal, differentiation, and migratory capacity of MSCs [59]. A study observing the effect of IGF-1 on the central nervous system showed that IGF-1 concentrations increase in the core and striatum of induced ischemic brain injury 48 h after MSC IV infusion [148]. MSC treatment also induced astrocytes to further enhance IGF-1 expression without increasing the number of activated astrocytes. The study suggests that IGF-1 may mediate a positive feedback loop in the regulation of astrocytes. Not only astrocyte-derived but also blood-derived IGF-1 was increased by MSC treatment on days 2, 4, and day 7. Since IGF-1 passes the BBB, the results suggest that MSC-treatment-mediated changes in IGF-1 levels in the blood may be an important mechanism leading to an increased level of IGF-1 in the brain [148]. Therefore, through their endocrine function mediation, MSCs exert a mitotic effect.

Proving the proliferative potential of IGF-1 in a renal model, blocking function with a specific antibody attenuated cell proliferation of cisplatin-injured proximal tubule epithelial cells (PTECs). When the PTECs were co-cultured with BM-MSCs, the MSCs provided a protective effect by promoting tubular cell proliferation via IGF-1 [149].

An important mitogenic autocrine impact of IGF-1 on MSCs is seen in genetically modified synovial-derived MSCs that overexpress IGF-1. These IGF-1-MSCs were superior to unmodified MSCs in chondrogenic differentiation potential without induction of the hypertrophic phenotype [150]. 

Mitotic paracrine effects of therapeutic MSC application have been shown to elicit their curative properties in various ways. However, whether they contribute to better adaptation of the tissue to stress/use, defect repair, stimulation of cellular proliferation into desirable phenotypes/cell types, or similar is still to be defined.

#### 3.1.2. Antimicrobial Effects

MSCs exhibit their antimicrobial effect via molecule secretion and direct cell-to-cell mechanisms, more precisely, the secretion of antimicrobial peptides and proteins (AMPs). AMPs that are responsible for antimicrobial activities are b-defensins (hBD-1, hBD-2, hBD-3), hepcidin, and lipocalin families (Lcn2) [151,152]. MSCs exert their antimicrobial activity through upregulation of LL-37, which is enhanced by bacterial stimuli and has been shown to reduce bacterial growth [153]. MSCs stimulated by bacterial lipopolysaccharide (LPS) can enhance antimicrobial functions of neutrophils, IL-8, and macrophage migration inhibitory factor (MIF), thus aiding the resolution of inflammation and infection [154].

#### 3.1.3. Immunomodulatory Effects

MSCs, in addition to trophic and antimicrobial effects, have also shown immunomodulatory properties [155]. Toll-like receptors (TLRs), expressed by MSCs, are responsible for sensing damage signals that activate the immunomodulatory response [156]. Their expression varies depending on tissue origin [157,158]. MSCs’ immunomodulation is exerted through paracrine activity as well as cell-to-cell contact through a wide range of bioactive molecules, including cytokines, chemokines, growth factors, etc., affecting both innate and adaptive immunity [159]. 

An example of these bioactive molecules is indoleamine 2,3-dioxygenase (IDO), an enzyme that catalyzes tryptophan into tryptophan metabolites, leading to tryptophan depletion [160]. It is secreted by MSCs upon IFN-γ stimulation, and its effects are enhanced by PGE2 [156]. Activating stress response kinase general control nonderepressible 2 (GCN2) through tryptophan reduction leads to cell cycle arrest in CD8+ T cells, blocks Th17 differentiation in CD4+ T cells, and promotes de novo Treg differentiation [161,162,163]. IDO, derived from BM-MSCs, has an inhibitory effect on natural killer (NK) cells, reducing their proliferation as well as cytolytic function and cytokine production [158,159,164]. Tryptophan reduction followed by increased IDO activity can also affect dendritic cells (DCs), leading to their higher expression of immunoglobulin-like transcript 3 (ILT3), resulting in an enhanced capacity to induce CD4+CD25+Foxp3+ Tregs [165]. BM-MSCs-derived IDO is responsible for the transformation of macrophages from the pro-inflammatory M1 phenotype, which secrete interleukin 1 (IL-1) and TNF-α, to the anti-inflammatory M2 phenotype, which secrete interleukin 10 (IL-10), IL-RA, and TGF-β [151]. Higher expression of IL-10 by M2 macrophages has an immunosuppressive effect on effector T cells [166].

Prostaglandin E2 (PGE2) is a lipid intermediate from arachidonic acid produced by cyclooxygenase-1 (COX-1) and cyclooxygenase-2 (COX-2), enzymes that are commonly expressed by MSCs [167]. Although PGE2 is synthesized by both BM-MSCs and AD-MSCs, basal level synthesis is significantly higher in AD-MSCs [168]. PGE2 interacts with receptors EP2 and EP4 exhibited on immune cells that, through activation of adenylate cyclase, increase the levels of cellular cyclic AMP (cAMP), leading to the expression of anti-inflammatory cytokines (IL-4, IL-5, IL-10) and inhibition of pro-inflammatory cytokines in an IL-2-dependent manner [169]. It also inhibits the production of IFN-γ in NK cells and promotes the production of TGF-β by myeloid-derived suppressor cells (MDSCs) [164,170]. PGE2 is responsible for M2 macrophage polarization [159]. Regarding T cells, PGE2 also exerts an immunosuppressive effect by promoting the Th-2 phenotype and Foxp3+ Treg cells and inhibiting the Th-1 phenotype [167,171]. By binding to the EP4 receptor on DCs, BM-MSCS-derived PGE2 inhibits DC differentiation and function [172].

Another cytokine that is commonly expressed by MSCs of different origins, such as BM-MSCs or AD-MSCs, is transforming growth factor (TGF)-β. Its expression is upregulated by pro-inflammatory factors such as IFN-γ and TNF-α [156,158,167]. TGF-β, together with HGF, can lead to downregulation of cyclin D2 and upregulation of p27kip1 in T cells, which results in cell cycle arrest in the G1 phase, thus inhibiting T-cell proliferation [156,169]. TGF-β and other anti-inflammatory cytokines (IDO, PGE2, IL-10) can induce the polarization of macrophages to the M2 anti-inflammatory phenotype [169]. Apoptotic cells secrete IL-10 and TGF-β, thus establishing an immunosuppressive milieu that inhibits lipopolysaccharide (LPS)-stimulated macrophages from secreting proinflammatory factors IL-1β and TNF-α [161]. TGF-β1 and other anti-inflammatory cytokines (PGE2, IDO) have an inhibitory effect on NK cells, affecting their proliferation and function [158].

Expressed and secreted by MSCs originating from different types of tissue (human placenta, bone marrow, adipose tissue), HGF is an immunomodulatory cytokine [173,174]. When secreted by human-placenta-derived MSCs, it acts on CD14+ monocytes through c-Met (HGF receptor) to induce the production of IL-10 via the ERK1/2 pathway. These CD14+ monocytes modulate T-cell cytokine profiles from a Th1 to a Th2 anti-inflammatory phenotype [173]. 

MSCs extracted from a variety of sources, such as Wharton’s jelly, adipose tissue, dental pulp, placenta, and bone marrow, express human leukocyte antigen-G (HLA-G) [175]. HLA-G binds to the receptors on the surface of several immune cells, exerting its immunomodulatory effects such as reducing NK cell and cytotoxic T cells cytolysis, allogeneic T cell proliferation, and DC maturation [176]. HLA-G also inhibits Th1/Th17 cytokine secretion [177]. The HLA-G5 isoform induces a high-IL-10-secreting Th2 profile and is required for MSCs’ induction of CD4+CD25highFOXP3+ Treg cells as well as inhibition of IFN- γ secretion by NK cells [176]. 

Both BM-MSCs and AD-MSCs secrete anti-inflammatory cytokine IL-10 [169,178,179]. IL-10 has an anti-inflammatory effect by downregulating catabolic matrix metalloproteinase (MMP) production [130]. Together with other anti-inflammatory cytokines (TGF-β, IDO, PGE2), IL-10 promotes M2 polarization of macrophages [180]. MSCs also promote IL-10-producing T cells via upregulation of CD210 [181]. IL-10 can also lead to T cell anergy by inhibiting CD28 expression by T cells and CD80/86 by DCs [182]. MSC-derived IL-10 induces regulatory DC differentiation as well [183]. Other anti-inflammatory properties of IL-10 are stimulation of macrophages to synthesize IL-1β antagonists, as well as inhibition of TNF-α, IL-6, and IL-12 [178]. IL-10 and HLA-G5 are linked in an amplification feedback loop, mutually increasing their concentrations and anti-inflammatory effect [176]. Regarding B cells, MSCs’ upregulation of IL-10 induces regulatory CD23+CD43+ B cells, which inhibit secretion of pro-inflammatory cytokines and T cell proliferation via an IL-10-dependent pathway [169].

An important factor of MSCs’ immunomodulatory effects is tumor necrosis factor-stimulated gene 6 (TSG6), expressed by both BM-MSCs and AD-MSCs [184,185]. TSG6 enhances the expansion of regulatory T cells as well as inhibits neutrophil recruitment by directly modulating neutrophil adhesion to the endothelium [184]. TSG6 acts by enhancing the inhibition of MMPs [184,186]. By binding to CD44 on macrophages, it interferes with the TLR2/NF-κB pathway, leading to reduced infiltration of neutrophils [187]. Furthermore, TSG6 also binds to fragments of hyaluronan, therefore diminishing its pro-inflammatory effect [186]. 

Extracellular ATP is a pro-inflammatory nucleoside, while its downstream hydrolyzation product adenosine (ADO) has anti-inflammatory properties [188]. Through the CD-39/CD-73/ADO pathway, MSCs expressing CD-73, together with activated T cells expressing CD-39, exert their immunomodulatory effects, such as inhibition of platelet aggregation and activation, T cell activation, reduction of NK cell activity, and induction of CD-73+ NK cells (which can regulate the function of NK cells, rendering them quiescent) [151]. 

Soluble proteins called galectins (Gal) bind to cell surface glycoproteins and are expressed by both BM-MSCs and AD-MSCs [156,189,190]. Galectin-1 regulates DC migration, signaling, and differentiation, as well as inhibits lymphocyte and neutrophil migration into inflamed tissues [191]. Galectin-1, together with Semaphorin-3A (Sema-3A), binds to a receptor expressed on T cells, neuropilin-1 (NP-1), inhibits T cell proliferation, and thus dampens inflammation [189]. Galectin-9 binds specifically to Tim-3, the Th1 cell surface receptor, inducing Th1 cell death (IFN-γ secreting cells) [192]. Galectin-9 can also impair B-cell proliferation and activity [193]. 

Another chemokine with immunomodulatory effects, C–C motif chemokine ligand 2 (CCL2), along with metalloproteinases responsible for its cleavage, is expressed by MSCs. Truncated CCL2 leads to inhibition of activated Th1, Th17, and NK cell migration to the site of inflammation [156]. BM-MSC-derived CCL2 inhibits the expression of transcription factor signal transducer and activator of transcription 3 (STAT3) which results in induction of paired box 5 (PAX-5) protein synthesis, thus inhibiting plasma cells from secreting immunoglobulin and limiting pathological humoral response [194]. 

Heme oxygenase-1 (HO-1) is a bioactive molecule that mediates the immunosuppressive effects of BM-MSCs and AD-MSCs [195,196]. HO-1, pharmacologically induced by cobalt protoporphyrin IX (CoPP), significantly decreased pro-inflammatory cytokines such as IL-1β, TNF-α, and IL-6 and impaired MMP-9 activity in a mouse model of non-autoimmune arthritis [197]. The anti-inflammatory effect of IL-10, a cytokine that induces HO-1 expression, is significantly reduced with HO-1 inhibition, contributing to HO-1’s role as IL-10 downstream mediator [198]. HO-1, derived from BM-MSCs, exerts its anti-inflammatory effect by inducing IL-10, Tr1, and TGF- β+ Th3 Treg proliferation [199]. 

IL-1 receptor antagonist (IL-1Ra) binds to IL-1R, thus blocking the pro-inflammatory effect of the IL-1/IL-1R pathway. These events include chondrocyte apoptosis, MMPs synthesis, and pro-inflammatory chemokine release [163]. Hence, by secreting IL-1Ra, BM-MSCs and AD-MSCs have anti-inflammatory and chondroprotective properties [200,201]. IL-1Ra is also responsible for M2 phenotype polarization in addition to IL-10 producing Breg-like cell generation, which leads to inhibition of plasmablasts and subsequent B cell differentiation [200]. 

Under TNF-α and IFN-γ stimulation, MSCs express and secrete programmed cell death ligand 1 (PD-L1) and programmed cell death ligand 2 (PD-L2) [202,203]. Interaction between PD-L1 on the surface of BM-MSCs and PD-1 on B cells leads to the inhibition of B cell proliferation and plasma cell differentiation [202]. Interaction of PD-L1 and PD-L2 with receptor PD-1 expressed on the surface of T cells leads to inhibition of activated CD4+ T cells and downregulation of IL-2 [204]. BM-MSCs and AD-MSCs upregulate the expression of PD-1 on T cells, thus leading to increased apoptosis of T cells [203,205]. 

Another molecular pathway, Fas/FasL, has an important mechanism in both BM-MSCs and AD-MSCs. While in AD-MSCs it has a proliferative role, in BM-MSCs it shows immunomodulatory properties [206,207]. The Fas/FasL pathway activated in BM-MSCs can induce T cell apoptosis. Decidual-derived MSCs have been demonstrated to have a distinct chemokine expression profile that has been hypothesized to account for their ability to modulate the functions of immune cells due to the presence of many chemokines that are known for their immunomodulatory effects, such as MCP-1 [208]. MSCs secrete Fas-mediated monocyte chemotactic protein 1 (MCP-1), which recruits T cells and then induces FasL-mediated apoptosis. Debris of apoptotic T cells is engulfed by macrophages, which are then stimulated to secrete TGF-β, leading to upregulation of regulatory T cells [206]. 

Further examination of the functions exerted by MSCs on the lymphocyte population was done by Li et al. Epithelial cells extracted from the amniotic portion of the placenta were shown to have an immunomodulatory effect in animal models. It was also observed that amniotic-cell-derived supernatants inhibit the chemotactic ability of neutrophils and macrophages through the inhibition of macrophage inflammatory protein (MIP-2). Furthermore, a caspase-3 assay concluded that the supernatant of the amniotic cells induced apoptosis of T and B cells [43]. The Jagged-1/Notch-1 signal pathway is another molecular pathway that is activated by BM-MSCs and promotes regulatory T cell differentiation, thus exerting an immunomodulatory effect [169].

STC-1, a glycoprotein hormone secreted by BM-MSCs, which also exerts trophic effects when upregulated, can have an anti-inflammatory effect and a protective role in OA by suppressing IL-6, IL-8, and MMP3/13 and inhibiting proliferation of OA-fibroblast-like synovial cells [51,209]. 

Adhesion molecules intercellular adhesion molecule-1 (ICAM-1) and vascular cell adhesion molecule-1 (VCAM-1), expressed by MSCs, increase their immunosuppressive effect on T cells by increasing cell–cell adhesion between both BM-MSCs and AD-MSCs and T cells [169,210].

Through the plethora of molecules mentioned, MSCs exert their anti-inflammatory effect, thus alleviating symptoms, reducing inflammation-induced cartilage damage, and aiding in cartilage repair in OA. However, to fully understand the underlying mechanisms and improve therapeutic methods, further investigation will be required.

### 3.2. Stromal Vascular Fraction and Microfragmented Adipose Tissue

Although AD-MSCs are the most readily available MSCs due to a more accessible extraction procedure than other tissues such as bone marrow or placental tissue, MSC isolation from harvested adipose tissue and MSC culture can be a problem in everyday practice and thus prevent the wider use of such therapeutic solutions.

For this reason, several new therapeutic options have emerged, such as the use of stromal vascular fraction (SVF) and microfragmented adipose tissue (MFAT), which avoid the need for further isolation and culturing of MSCs. MSCs are therefore contained in an environment that is not purely made of MSCs but also pre-adipocytes, endothelial cells, endothelial precursor cells, macrophages, smooth muscle cells, lymphocytes, and pericytes [211]. Such protocols have allowed wider use of MSC-based therapies while retaining immunomodulatory, anti-inflammatory, and angiogenic functions that have shown to be crucial in the mechanism of action of MSCs. The use of SVF or MFAT over cultured MSCs theoretically offers several beneficial effects. One of them is the physical interaction between AD-MSCs and surrounding endothelial cells and extracellular matrix, which act as structural support for AD-MSCs. The mechanical process of AD-MSC acquisition can preserve cells in clusters and therefore maintain the native environment in order to sustain original cell function [212]. Furthermore, neighboring endothelial progenitor cells (EPCs) and other components of the ECM excrete numerous cytokines and growth factors, such as platelet-derived growth factor (PDGF)-BB, which stimulate AD-MSCS to proliferate and migrate to target areas [211,213]. Another important factor is the “homing effect” which represents the migration of AD-MSCs to the target tissue via interaction with chemokine receptors, integrins, selectins, etc. Moreover, the high number of passages during culture expansions that are required to obtain pure AD-MSCs are responsible for the loss of their homing effect. Such a scenario is not evident in the case of SVF and MFAT, which, therefore, leads to better adherence at the site of cartilage injury [213]. 

However, obtaining SVF requires digestion of lipoaspirate with collagenase, which can also create regulatory problems in certain countries. 

The necessary enzymatic reaction and prolonged ex vivo expansion during the preparation of SVF or ADMSCs derived from fat lipoaspirate have their unavoidable negative effects on regenerative potential, with consequential attenuation of clinical outcomes [30]. Enzymatic treatment is considered to destroy the ECM and damage cells and exosomes, affecting the viability of SVF [214]. Considering this, enzyme-free systems were developed for micro-fragmented adipose tissue (MFAT) preparation. This technology is based on the use of mild mechanical force to reduce the size of adipose tissue clusters while preserving vascular stroma, avoiding any enzymatic or other chemical treatment [215]. The aforementioned preservation of vascular architecture is responsible for the higher pericyte content and secretory activity of MFAT. MFAT has shown a higher secretion of growth factors, such as angiogenin, angiostatin/plasminogen, hepatocyte growth factor (HGF), placental growth factor (PlGF) endoglin, leptin, PDGF-AB/BB, thrombospondin 2, and uPA, than SVF. Furthermore, it has been found that MFAT secreted significantly higher values of adiponectin, CD14, CD31, CD40 ligand (CD154), TNF-α, complement factor D, uPAR (CD87), RANTES, MCSF, GDF-15, IGFBP-2, IL1RA, IP-10, MIG (CXCL9), MIP-3β (CCL19), PDGF-AA, RBP-4, and ST2, contributing to its immunomodulatory functions [216]. An intact perivascular niche was postulated to be a crucial factor in the high secretion of cytokines and growth factors [216]. In addition, MFAT was found to have potent angiogenic and anti-inflammatory effects. Anti-inflammatory properties were evaluated through inhibition of U937 migration upon stimulation with the chemokine MCP-1, suppression of their adhesion to HUVECs, and downregulation of RANTES and MCP-1 secretion [30]. 

Despite the promising results that SVF and MFAT techniques provide, it is necessary to further clarify the role of cells that surround the MSCs, more specifically the perivascular niche that remains intact in MFAT, and how the presence of these cells affects the behavior of MSCs.

## 4. Clinical Results

Despite the fact that knee OA is a very common condition that affects 16% of the global population, its treatment remains challenging [212]. Pathogenesis of knee OA is still the subject of extensive research. Numerous therapeutic options based on the application of MSCs have been developed in the past few years. 

Several meta-analyses were conducted to evaluate the effect of MSCs and SVF on clinical and radiological symptoms of patients diagnosed with knee OA. Tan et al. investigated whether intra-articular injections of MSCs without adjuvant therapies are effective in the treatment of knee OA [217]. A comparison between post-intervention and preintervention clinical findings was conducted. A total of nine studies with 440 knees were included. Patients with additional injections, additives placed in their MSC injections, or adjuvant surgeries were excluded from the study. The results showed that MSC treatment significantly improved pain and function for knee OA. Specifically, VAS for pain, Western Ontario and McMaster Universities Osteoarthritis Index (WOMAC) total score, and Knee injury and Osteoarthritis Outcome Score (KOOS) showed improvements when comparing outcomes before and after therapeutic intervention. Furthermore, MSC origin varied between the studies included in the meta-analysis, so the authors compared the effect of MSCs isolated from different tissues. Significantly better clinical results were noted with the use of BM-MSCs compared to AD-MSCs [217].

On the other hand, a meta-analysis conducted by Han et al. showed superior therapeutic effects of AD-MSCs compared to BM-MSCs in the treatment of knee OA [218]. Patients treated with AD-MSCs showed significant improvements in VAS and WOMAC scores at 6-, 12-, and 24-month follow-ups. In contrast, patients treated with BM-MSCs showed VAS improvement at 6-, 12-, and 24-month follow-ups, but WOMAC showed no statistical significance, even though there was a positive trend during follow-up.

The placenta-derived MSCs and their therapeutic potential were examined in a double-blind, placebo-controlled clinical trial by Khalifeh Soltani et al. [219]. Twenty patients were divided into two groups to receive either placenta-derived MSCs or normal saline. Clinical and radiological improvements were followed up for 24 weeks. Knee joint range of motion (ROM) was significantly improved between week 2 and week 24, but VAS showed no improvement. KOOS indicated significant improvement in several subscales, but all of the effects were imperceivable 8 weeks post-injection. MRI was evaluated at a 24-week follow-up and showed chondral thickening in approximately 10% of total knee joint cartilage areas. Larger scale studies are needed to better define the clinical efficacy of placenta-derived MSC treatment and to compare it with more extensively researched MSC treatments such as AD-MSCs and BM-MSCs.

To better understand the therapeutic potential of MSC treatment in knee OA, it is necessary to compare it with placebo as well as other commonly used treatments, such as hyaluronic acid (HA) injections, platelet-rich plasma (PRP) injections, arthroscopic debridement, high tibial osteotomy (HTO), and conservative management.

Naja et al. performed a meta-analysis based on recent randomized controlled trials (RCTs) to quantify the efficacy of nonsurgical interventions commonly used in patients with mild to moderate knee OA [220]. Nineteen RCTs were included in this meta-analysis, and several treatment options, such as MSCs, PRP, HA, corticosteroids, NSAIDs, etc., were evaluated. The primary outcome was the mean change in WOMAC total score from baseline to 12 months. A significant decrease in WOMAC total score was observed in MSC and PRP intervention groups. MSC and PRP interventions showed the most consistent improvement in pain and articular function in the long term when compared with other interventions included in this study. However, MSC treatment showed the greatest improvement in pain reduction and gain in function, which could indicate its superiority over the other treatment options evaluated in this meta-analysis.

A comparison of the therapeutic effect of MSCs vs. HA was further examined in a meta-analysis by Jiang et al. [221]. HA injection was set as the control, while the treatment group received MSC injections. Results showed significant improvement in WOMAC-total and WOMAC-pain subscale at 6 and 12 months in the treatment group compared with the control group. However, RCTs included in this meta-analysis that evaluated MRI scores showed no statistically significant improvement in MRI scores at 6 and 12 months of follow-up. Furthermore, studies included in this meta-analysis lack uniformity in the methods of MSC preparation, dosage, and usage. Such heterogeneity of MSC-based procedures is precisely the reason why those treatment options could not find a place in the guidelines for OA management from the leading professional societies [2]. Therefore, even though intraarticular MSC therapy is a promising therapeutic option for patients suffering from knee OA, further research is needed to determine the best therapeutic protocol for MSC application.

Another emerging therapeutic option for the treatment of knee OA is the intra-articular application of autologous MFAT. Compared with MSC treatment, adipose stem cells (ASCs) are not expanded in vitro but extracted from the adipose tissue of the patient within the operating room [212]. As a result, such a protocol gives rise to several cell types not strictly limited to AD-MSCs. One study described cell types found in samples of stromal vascular fraction from lipoaspirate (SVF-LA) and stromal vascular fraction from microfragmented lipoaspirate (SVF-MLA) [222]. The most prominent population phenotypes were endothelial progenitor cells (EPC), endothelial mature cells, pericytes, transitional pericytes, and supra-adventitial-adipose stromal cells (SA-ASCs). It is important to note that EPCs outnumbered MSCs, which could indicate the involvement of EPCs in the overall effect of SVF treatment [222]. Anil et al. conducted a meta-analysis of randomized control trials to compare the efficacy of intra-articular injectable treatments in patients with knee OA [223]. Treatments evaluated in this study included autologous conditioned serum (ACS), bone marrow aspirate concentrate (BMAC), botulinum toxin, corticosteroids (CS), HA, MSCs, ozone, saline placebo, PRP, plasma rich in growth factor (PRGF), and SVF. The main finding of this study was that the SVF treatment resulted in the biggest reduction of VAS at all time points (4 weeks to 12 months) and for WOMAC at 12 months post-injection. It is important to note that the majority of the other intra-articular injections showed better clinical results than the saline placebo, which indicated their clinical efficacy. Therefore, a clear improvement in clinical outcomes is visible with MSC therapy.

Gobbi et al. evaluated clinical outcomes of autologous MFAT injection in patients with knee OA, with the primary outcome being KOOS, measured at baseline, 6, 12, and 24 months post-treatment [224]. The authors also calculated minimal clinically important difference (MCID) status for several KOOS subscales (pain, symptoms, ADLs) and VAS. A total of 120 primary treatments were assessed. Significant improvement in KOOS-Pain was observed at six months follow-up when compared with the baseline values and plateaued until 24 months post-treatment. A finding of interest was that about one in three patients did not exceed MCID for KOOS at the 24-month follow-up when compared with the pre-treatment score. Such individuals were characterized as non-responders, and the analysis showed higher baseline KOOS-ADL in this group, which indicated that patients with higher baseline functional demands may not respond as well to the treatment. As such, the patient’s functional demands should be taken into consideration when initiating MSC therapy. 

Another study examined the clinical effectiveness and safety of a single intra-articular injection of MFAT in OA patients [225]. The authors used VAS and KOOS for clinical evaluations conducted 6-, 12-, and 24-months post-treatment. A total of 202 patients were eligible for the study. Significant improvement in total KOOS was noted between baseline and 6-month follow-up and again between 6- and 12-months follow-up. At 24 months, there was no statistically significant improvement in the total KOOS score when compared with the results obtained at the 12-month follow-up, but the total KOOS score was still significantly improved when compared with the values obtained at the baseline and 6 months. Somewhat different results regarding the dynamics of the treatment were seen with VAS, as it showed a significant reduction at 6 months, but then it increased again at 12 months. A further increase in VAS was seen at 24 months, to the point of no significant difference compared to the baseline values. Interestingly, discrepancy between VAS and KOOS-Pain was noted, as the KOOS-Pain subscale showed significant improvement compared to the baseline at all time points. The authors also reported lower clinical scores in patients that underwent physical therapy or participated in sports activities after injection. Based on these observations, an effort should be made to determine the optimal rehabilitation strategy and avoid functional overload in patients treated with MFAT injections.

Further insight into the dynamics of cell-based treatments, such as SVF and MSCs, was provided in the meta-analysis by Agarwal et al. [226]. Studies included in this meta-analysis investigated the therapeutic potential of AD-MSCs and SVF. WOMAC scores were evaluated in each follow-up (up to 24 months), and the results showed significant improvement in every subsequent follow-up. A slight reduction in WOMAC scores was noted at 24 months when compared to 18 months. Overall, the results of this meta-analysis not only showed that both AD-MSC and SVF treatments provide significant relief of OA symptoms, but also emphasized that the greatest clinical effect of the treatment can be expected in the first 18–24 months post-treatment. Wei et al. conducted a meta-analysis in order to compare the efficacy of MSCs obtained from different sources (AD-MSCs, BM-MSCs, UC-MSCs) [227]. One of the outcomes of the study was cartilage regeneration, assessed by Whole-Organ Magnetic Resonance Imaging Score (WORMS) or another structural assessment scale if the WORMS was not reported in a specific study included in the meta-analysis. Furthermore, clinical findings were evaluated by VAS and WOMAC scores. The efficacy of MSCs from different sources was ranked based on the SUCRA score. Results showed that MSCs compared with HA or placebo did not achieve statistically significant improvement in cartilage regeneration. Despite such results, differences between MSCs obtained from distinct sources were noted, and AD-MSCs showed the highest score and therefore the highest probability of being the best treatment option for cartilage regeneration.

Similar findings were reported in a meta-analysis by Dai et al. that also included MRI WORMS score as one of the outcomes of the study [228]. A total of four studies (93 knees) provided MRI WORMS scores for the MSC-treated knees compared with either placebo or HA, as determined by specific studies included in the meta-analysis. No significant difference was observed between the MSC treatment group and the control groups.

MRI changes in patients with knee OA treated with MSCs were also evaluated in a meta-analysis by Ma et al., which analyzed WORMS score and changes in cartilage volume [229]. All studies included in this meta-analysis, which evaluated MRI changes, were followed up for 12 months. MSC groups showed significant improvement in cartilage volume when compared to the control groups, but the WORMS score showed no statistically significant changes between the two groups.

Several problems regarding MSC and SVF treatment in OA still exist, one of them being high heterogeneity in obtaining MSC and SVF formulations and application protocols. Furthermore, current evidence shows inconsistent and often conflicting results in terms of the clinical and radiologic benefits of such treatments. Overall, MSC and SVF therapy show promising results, but further research is needed to determine the exact role of these treatment options for knee OA.

## 5. Conclusions

The pathophysiology of OA as a disease is well defined; however, discrepancies in potential MSC treatment remain. Bridging the pathologic processes and research must be better applied. The exact ratios or quantities of molecules and growth factors in MSC use remain unquantified. While the current body of evidence for MSCs is vastly growing, there exists no standardization in extraction and, even more importantly, no descriptive protocol in previously conducted research. A treatment method without well-defined parameters will never be included in standard practice protocols. That is why better quantification of such molecules, followed by standardization in extraction methods, will lead to concrete conclusions and to the adoption of such treatment methods in current guidelines. Therefore, more research on the paracrine and autocrine exosomes and their application to osteoarthritic pathologies must be done in a uniform manner with well-defined parameters and markers.
